# Clinical trials registries: is it viable for the inclusion of conduct, performance, analyses and cost of trials?

**DOI:** 10.1186/1745-6215-14-S1-O14

**Published:** 2013-11-29

**Authors:** Amanda Young, James Raftery, Louise Stanton, Andrew Cook, Peter Davidson, Ruairidh Milne, David Turner

**Affiliations:** 1 National Institute for Health Research, Evaluation, Trials and Studies Coordinating Centre, Southampton, UK; 2 Clinical Trials Unit, University of Southampton, Southampton, UK; 3 Wessex Institute, University of Southampton, Southampton, UK; 4 Faculty of Medicine and Health Sciences, University of East Anglia, East Anglia, UK

## Background

In the UK randomised clinical trials mainly register with the ISRTN and ClinGov. However, the quality of reporting is poor. No studies have identified moving beyond the minimum data set for prospective registration to include conduct, performance, cost and results of trials.

## Objectives

To test the feasibility of specified questions under six themes (origin of topic, trial conduct and performance, statistical and economic analyses, and trial costs). To pilot a database structured around those questions.

## Methods

We assessed the NIHR HTA portfolio for all published randomised clinical trials from 1999 to 2011.

The feasibility element explored the operationalisation of 85 proposed questions. Each question was assessed for data availability, time needed to extract and analyse data. Questions deemed feasible were eligible for full data extraction in the pilot study.

## Results

109 HTA funded projects published in the HTA Journal Series met inclusion criteria (a randomised clinical trial). Of the 85 original questions, seven were deemed not feasible. 78 questions were eligible for the pilot study. Each question was judged on completeness, amendments, skills and resource. Of the 78 questions, 33 were recommended to ‘keep', 28 for ‘amend' and 17 to ‘drop'.

## Conclusions

Our findings suggest that it is feasible to move beyond a limited minimum dataset. Extending the database to include all NIHR clinical trials could help to better understand the conduct, performance, analyses and cost of trials. To maximise the added value of their funding of trials with high quality science, metadata on those trials is essential.

